# The reduction in CD8^+^PD-1^+^ T cells in liver histological tissue is related to Pegylated IFN-α therapy outcomes in chronic hepatitis B patients

**DOI:** 10.1186/s12879-020-05320-z

**Published:** 2020-08-10

**Authors:** Ruyu Liu, Yanhui Chen, Jiang Guo, Minghui Li, Yao Lu, Lu Zhang, Ge Shen, Shuling Wu, Min Chang, Leiping Hu, Hongxiao Hao, Henghui Zhang, Yao Xie

**Affiliations:** 1 Department of Hepatology Division 2, Beijing Ditan Hospital, Capital Medical University, No. 8 Jingshun East Street, Chaoyang District, Beijing, 100015 China; 2 Institute of Infectious Diseases, Beijing Ditan Hospital, Beijing Key Laboratory of Emerging Infectious Diseases, Capital Medical University, No. 8 Jingshun East Street, Chaoyang District, Beijing, 100015 China; 3 Genecast Precision Medicine Technology Institute, 35 North Garden Rd., Haidian District, Beijing, 100089 China; 4 Tumor Interventional Department, Beijing Ditan Hospital, Capital Medical University, No. 8 Jingshun East Street, Chaoyang District, Beijing, 100015 China

**Keywords:** Chronic hepatitis B, Pegylated IFN-α, CD8^+^ T cells, CD4^+^ T cells, CD68^+^ mononuclear cells, PD-1

## Abstract

**Background:**

Antiviral therapy is recommended for patients with immune-active chronic hepatitis B (CHB) to decrease the risk of liver-related complications. However, the outcomes of the pegylated IFN-α (PEG-IFN-α) therapy vary among CHB patients. We aimed to identify factors that can influence the outcomes in CHB patients who received antiviral PEG-IFN-α monotherapy.

**Methods:**

Thirty-two CHB patients who received PEG-IFN-α monotherapy were enrolled in this study. All of the patients underwent two liver biopsies at baseline and 6 months after the initiation of the therapy. CD8^+^ T cells, CD4^+^ T cells, CD68^+^ mononuclear cells, and PD-1 levels in the 64 liver biopsy specimens were examined via immunofluorescence.

**Results:**

The overall median frequency of CD8^+^ T cells in the liver tissues of 32 CHB patients significantly decreased at 6 months after the therapy initiation (*p* < 0.01). In the FIER (fibrosis and inflammation response with HBeAg seroconversion) group, CD8^+^PD-1^+^ T cells significantly decreased at 6 months (*p* < 0.05), while CD8^+^PD-1^−^ T cells had no significant difference. On the contrary, in the FIENR (no fibrosis and inflammation response and HBeAg seroconversion) group, CD8^+^PD-1^−^ T cells significantly decreased after 6 months of PEG-IFN-α treatment (*p* < 0.05), while CD8^+^PD-1^+^ T cells had no significant difference. In addition, the levels of CD68^+^ mononuclear cells in the FIER group showed an overall increasing trend after treatment (*p* < 0.05).

**Conclusions:**

The changes in the levels of CD8^+^PD-1^+^ T cells and CD68^+^ mononuclear cells may be related to the response to PEG-IFN-α therapy.

## Background

It is estimated that 3.9% of the global population is positive for hepatitis B s-antigen (HBsAg), which corresponds to 291,992,000 infections in total [1]. Approximately, chronic HBV infection-related diseases including liver cirrhosis and hepatocellular carcinoma (HCC) will kill about 786,000 people annually in the world [2]. To reduce such risk of death-causing complications, antiviral therapy is highly recommended for patients with immune-active chronic hepatitis B (CHB) [3, 4]. Currently, peginterferon along with nucleos(t)ide analogs (NAs), such as tenofovir alafenamide (TAF), tenofovir disoproxil fumarate (TDF), and entecavir (ETV), are the preferred antiviral agents in clinic [3, 4]. As a first-line drug for the treatment of chronic hepatitis B infection, PEG-IFN-α mediates the antiviral, antiproliferative, and immunomodulatory effects [5]. However, its efficacy is limited in only one-third of treated patients [3, 6].

IFN-α is a multifaceted cytokine that functions in both direct antiviral activity and immunomodulation. IFN-α induces the expression of hundreds of interferon-stimulated genes (ISGs), many with antiviral effector functions [7]. On the other hand, it can activate NK cells, dendritic cells, and B cells, as well as the T cell functions through both direct and indirect ways [7–9]. IFN-α has been shown to play an important role in the differentiation of both CD4^+^ and CD8^+^ T cells [8]. It was shown that PEG-IFN-α therapy led to a striking reduction in CD8^+^ T cells when treating CHB patients [10, 11]. However, changes in the opposite direction was also reported and with no difference in CD4^+^ T lymphocytes in the liver tissue of CHB patients [12]. Although IFN-α has been used for over 20 years in clinic to treat CHB infections, it remain in mysterious what factors determine the responsiveness to it.

Extended upregulation of PD-1 is associated with T cell exhaustion and persistent viral infection [13]. Our past study has demonstrated that in PEG-IFN-α therapy responders, PD-1 levels decreased in CD4^+^ and CD8^+^ T cells from peripheral blood, indicating that PD-1 expression in CD4^+^ and CD8^+^ T cell might influence the outcomes of PEG-IFN-α therapy [6]. Whether this is the same in liver tissues of CHB patients should be confirmed. In liver tissue, CD68^+^ mononuclear cells were regarded as Kupffer cells (KCs) and liver-infiltrating monocytes/macrophages [14, 15]. Studies showed that these cells could induce pro-inflammatory response, which is important to inhibit viral replication [16]. However, the interaction between KCs and virus can also prohibit effective viral immunity, facilitate viral persistence, and promote liver damage [16]. Furthermore, pro-fibrinogenic factors secreted by KCs may stimulate fibrogenesis [16]. Recent studies have demonstrated that liver-resident macrophages only contributed to a small fraction of those effects, while the major role was played by macrophages formed from newly recruited monocytes in the inflamed and damaged liver [17, 18]. Nevertheless, the changes in CD68^+^ mononuclear cells in liver tissue during PEG-IFN-α therapy are not fully addressed.

In this study, we assessed the changes in CD4^+^ T, CD8^+^ T, and CD68^+^ mononuclear cells and PD-1 levels in liver tissue at baseline and 6 months after the initiation of PEG-IFN-α monotherapy in 32 CHB patients. We further analyzed the differences between responders and non-responders in order to find the baseline and on-treatment factors that may influence the PEG-IFN-α therapeutic outcomes.

## Methods

### Patients

A total of 32 HBeAg-positive CHB patients were enrolled in this retrospective study from December 2008 to September 2011 at Beijing Ditan Hospital, Beijing, China. All of the patients were positive for HBsAg for more than 6 months, and received 180 μg of PEG-IFN-α weekly for 1 year. The biochemical, serological, and virological parameters were measured at baseline and at 3, 6, 9, and 12 months after the initiation of the regimen to monitor the efficacy and any side effects. Liver biopsies were performed at baseline and 6 months after the initiation of the regimen in all the patients to evaluate the liver fibrosis stage, histologic activity, and the expression of CD8, CD4, CD68, and PD1. The exclusion criteria of this study were (i) co-infection with hepatitis C, D, or human immunodeficiency virus; (ii) HCC development after the therapy commencement; and (iii) lack of compliance with PEG-IFN-α treatment for more than 3 months. This study was approved by the Institutional Review Board of Beijing Ditan Hospital (Reference number 161508), and informed consent was signed.

### Laboratory data

The biochemical, serological, and virological parameters were measured using standard laboratory procedures. HBV serology included HBsAg, anti-HBs, hepatitis B e-antigen (HBeAg), anti-HBe, and anti-HBc tests. Serum HBsAg test was performed using commercial kits (Abbott Laboratories; Lake Bluff, IL, USA). Serum HBV DNA levels were assessed via real-time polymerase chain reactions (COBAS TaqMan HBV Test v2.0, Roche Diagnostics, Branchburg, NJ, USA). A serum HBV DNA level at 20 IU/mL was set as the threshold for HBV-DNA positivity. HBeAg seroconversion was defined by the disappearance of HBeAg with or without detectable anti-HBe.

### Liver histological assessment

Percutaneous liver biopsies were performed, and paraffin sections were prepared according to a previous study [19]. The biopsy samples were assessed by two independent pathologists who were blinded to the results of noninvasive fibrosis tests. Discordant cases were reviewed by a third highly experienced pathologist. The liver fibrosis stage and histologic activity were determined by METAVIR scoring system, which has been adopted as the pathological diagnosis standard of liver inflammation and fibrosis. Liver inflammation was divided into four grades: A0, no inflammation; A1, mild inflammation (focal, few portal areas); A2, moderate inflammation (most portal areas, and even extending beyond the portal areas); and A3, severe inflammation (significant confluent necrosis and bridging necrosis). Liver fibrosis was divided into five stages: F0, no fibrosis; F1, portal fibrosis without septa; F2, portal fibrosis with rare septa; F3, numerous septa without cirrhosis; and F4, cirrhosis [19]. An inflammation response was defined as a decrease in the METAVIR inflammation score ≥ 2 points from baseline and no worsening of the fibrosis score. Fibrosis response was defined as a decrease in the METAVIR fibrosis score no less than 1 point from baseline.

### Multiplex immunohistochemical staining

**Fig. 1:**
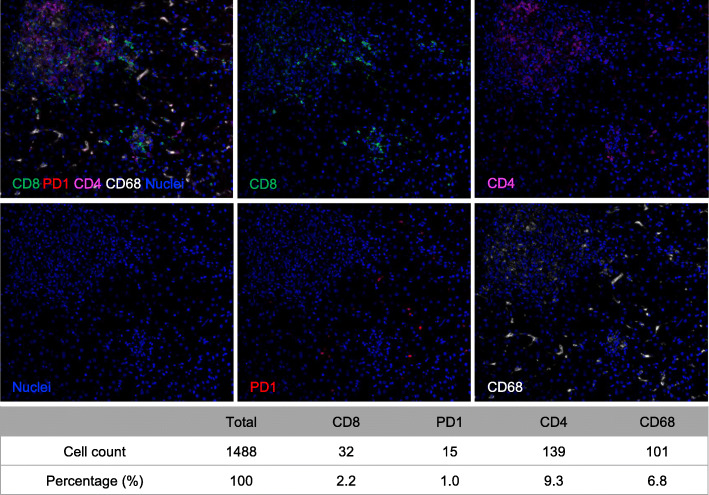
Representative images of IHC staining. Representative IHC staining images from case 232,643, who belongs to the FIER group. On the same slice, different proteins and nuclei were labeled with different fluorescence. CD8^+^ T cells are in green. PD-1^+^ cells are in red. CD4^+^ T cells are in magenta. CD68^+^ mononuclear cells are in white. Nuclei are in blue. 100× magnification. The table gives the numbers and corresponding percentages of different types of cells

Paraffin-embedded tissue blocks were sectioned into 3 μm sections. The slides were deparaffinized in xylene, rehydrated, and washed in tap water before boiling in Tris-EDTA buffer (pH 9; 643,901; Klinipath) for epitope retrieval/microwave treatment (MWT). Endogenous peroxidase and protein blocking were performed using Antibody Diluent/Block (72,424,205, PerkinElmer) for 10 min at room temperature. In this study, there were two detection panels. One includes CD4, CD8, CD68, and PD-1 and the other includes CD68, CD49A, and MARCO. The antigens were labelled sequentially. Each round of antigen labelling consisted of three steps: primary antibody incubation, secondary antibody incubation, and tyramide signal amplification (TSA) visualization. The complexes of antigen-labelled primary/secondary antibody and TSA were removed by MWT with Tris-EDTA buffer (pH 9) at the end of each round of labelling, and then the next round of antigen labelling was performed on the same slice following the procedures mentioned above. The labelling was finished with MWT, counterstained with DAPI for 5 min, and mounted in Antifade Mounting Medium (I0052, NobleRyder). The primary antibodies used were anti-CD4 antibody (ZM-0418, clone UMAB64; Zsbio, dilution 1:100), anti-CD8 antibody (ZA-0508, clone EP334; Zsbio, dilution 1:100), anti-CD68 antibody (ZA-0060, clone KP1; Zsbio, dilution 1:400), anti-PD-1 antibody (ZM-0381, clone UMAB199; Zsbio, dilution 1:100), anti-CD49A antibody (ab243032, clone CL7207; Abcam, dilution 1:100) and anti-MARCO antibody (ab231046; Abcam, dilution 1:100). The primary antibodies were incubated according to the manufacturer’s instructions. Incubation with Polymer HRP Rb (PV-6001, Zsbio) or Polymer HRP Ms. (PV-6002, Zsbio) was performed at 37 °C for 10 min. TSA visualization was performed with an Opal seven-color IHC Kit (NEL797B001KT, PerkinElmer) containing fluorophores DAPI, Opal 520 (for CD49A), Opal 570 (for CD4 or CD68), Opal 620 (for CD8), Opal 650 (for CD68 or MARCO), Opal 690 (for PD-1), and TSA Coumarin system (NEL703001KT, PerkinElmer). Representative IHC staining results are shown in Fig. 1.

### Tissue imaging and analysis

The slides were scanned using PerkinElmer Vectra (Vectra 3.0.5, PerkinElmer). Multispectral images were unmixed using spectral libraries built from images of single stained tissues for each reagent using inForm Advanced Image Analysis software (inForm 2.3.0, PerkinElmer). A selection of 5–15 representative original multispectral images was used to calibrate the inForm software (tissue segmentation, cell segmentation, phenotyping tool, and positivity score). All of the settings applied to the calibration images were saved in an algorithm to allow batch analysis of multiple original multispectral images of the same tissue. Positive cells were defined as cells with true immunofluorescence signal detected and with right expression pattern. The expression of CD8, CD4, CD68, and PD-1 was reported as a continuous variable with the percentage of positive cells stained with any intensity (Fig. 1). Two pathologists confirmed the quality and results of the experiment.

### Statistical analysis

Statistical analyses were conducted using GraphPad Prism (version 8.2.0, La Jolla, CA, USA) and SPSS version 22.0 (SPSS, Inc., Chicago, IL, USA). The results are as follows: normal distribution data as mean ± SD, non-normal distribution continuous data as median (interquartile range), and categorical variables as number (percentage). The *t* test, χ^2^, or Mann-Whitney test were performed to determine the significance of differences between different groups. Statistical significance was accepted for *p*-values < 0.05. One asterisk (*) represents *p* < 0.05 and double asterisks (**) represent *p* < 0.01.

## Results

### Characteristics at baseline and improvement after 6 months of PEG-IFN-α treatment

The characteristics of all 32 patients at baseline, and 3, 6, 9, and 12 months after the initiation of PEG-IFN-α therapy are shown in Table 1. A total of 24 (75%) patients were male, and 8 were female (25%). According to the METAVIR scoring system, the numbers of patients at each METAVIR fibrosis stage were as follows: 18 (56.3%) at F1, 9 (28.1%) at F2, 4 (12.5%) at F3, and 1 (3.1%) at F4. The numbers of patients in each METAVIR inflammation group were as follows: 6 (18.75%) in A1, 14 (43.75%) in A2, and 12 (37.5%) in A3. After 6 months of PEG-IFN-α treatment, the levels of WBC, RBC, PLT, ALT, AST, TBIL, HBV-DNA, and HBeAg decreased significantly (*p* < 0.05) in total (Table 1). The rates of patients with normal ALT, normal AST, and HBV-DNA negative increased during the course of PEG-IFN-α therapy (Table 2). In this study, 43.8% (14/32) patients achieved a fibrosis response and 68.8% (22/32) achieved an inflammation response (Table 2). The means of scoring fibrosis and inflammation stages at baseline were 1.63 ± 0.83 and 2.19 ± 0.74, respectively. Those numbers significantly decreased to 1.19 ± 0.86 and 1.5 ± 0.72, respectively (*p* < 0.05) (Table 3) at 6 months.

**Table 1:** Characteristics of the CHB patients

Characteristics	Baseline	3 months	6 months	9 months	12 months
Age (month, M ± SD)	364.03 ± 92.12	367.03 ± 92.12	370.03 ± 92.12	373.03 ± 92.12	376.03 ± 92.12
Gender (male/female)	24/8	24/8	24/8	24/8	24/8
WBC count (10^9^/L)	5.69 ± 1.48	4.14 ± 1.18	3.64 ± 1.01^**^	3.52 ± 1.14^**^	3.33 ± 1.13^**^
RBC count (10^12^/L)	4.87 ± 0.64	4.83 ± 0.55	4.61 ± 0.64	4.55 ± 0.60^*^	4.48 ± 0.63^*^
Platelet count (10^9^/L)	190.74 ± 60.21	144.86 ± 51.92^**^	122.69 ± 44.02^**^	118.42 ± 47.74^**^	113.16 ± 45.33^**^
ALT, U/L (median)	98.95 (74.8–189.48)	85.85 (68.5–116.33)	66.2 (44.9–118)^**^	58.35 (39.05–73.98)^**^	46.4 (32.58–56.83) ^**^
AST, U/L (median)	57.8 (44.25–83.95)	51.05 (36.4–65.78)	43.9 (35.2–58)^*^	36.3 (28.15–45.73)^**^	29.8 (23.35–39.05) ^**^
TBIL, (umol/L, M ± SD)	18.7 ± 7.61	17.93 ± 9.24	15.04 ± 9.39	14.72 ± 8.41	13.74 ± 7.32^*^
DBIL, (umol/L, M ± SD)	5.62 ± 3.7	6.36 ± 4.99	5.32 ± 3.34	5.44 ± 3.16	4.95 ± 2.95
ALB, (g/L, M ± SD)	45.71 ± 4.9	45.53 ± 4.98	45.66 ± 4.7	45.93 ± 4.46	45.97 ± 4.65
HBeAg (median)	864.31 (362.12–1236.09)	456.83 (103.55–1210.75)	395.27 (0.56–996.45)^*^	93.77 (0.38–1020.25) ^**^	52.86 (0.29–1054.42) ^**^
HBeAg positive/negative	32/0	29/3	22/10	19/13	17/15
HBV-DNA (log10) (median)	7.21 (6.7–7.73)	6.74 (4.6–7.8)	6.29 (3.51–7.33)^*^	5.4 (2.7–6.35) ^**^	3.95 (2.67–5.94) ^**^

^*^ Represents *p* < 0.05, ^**^ represents *p* < 0.01

**Table 2:** Changes in response indicators

Characteristics	Antiviral therapy		
	Baseline	6 months	12 months
ALT normal/total [n (%)]	3/32 (9.4%)	12/32 (37.5%)	21/32 (65.6%)
AST normal/total [n (%)]	5/32 (15.6%)	10/32 (31.3%)	25/32 (78.1%)
HBV-DNA negative [n (%)]	0/32 (0%)	6/32 (18.8%)	10/32 (31.3%)
HBeAg seroconversion [n (%)]	0/32 (0%)	10/32 (31.3%)^*^	15/32 (46.9%)
Fibrosis improvement [n (%)]	0/32 (0%)	14/32 (43.8%)	NA
Inflammation improvement [n (%)]	0/32 (0%)	22/32 (68.8%)	NA

^*^ Represents *p* < 0.05. NA represents not available. A serum HBV-DNA level < 20 IU/mL was defined as the limit of detectability

**Table 3:** Liver tissue characteristics of PEG-IFN-α treated CHB patients

Characteristics	Baseline	6 months
Fibrosis stage (METAVIR)	1.63 ± 0.83	1.19 ± 0.86*
Inflammation stage (METAVIR)	2.19 ± 0.74	1.5 ± 0.72*
CD4^+^ T cells (%) (median)	16.5 (10.16–23.94)	12.55 (7.6–19)
CD8^+^ T cells (%) (median)	2.18 (1.23–3.98)	0.62 (0.27–2.46)**
CD68^+^ cells (%) (median)	1.39 (0.61–3.63)	1.16 (0.16–4.36)
PD-1^+^ cells (%) (median)	0.5 (0.10–0.90)	0.29 (0.08–0.64)

^*^ Represents *p* < 0.05, ^**^ represents *p* < 0.01

### CD8^+^PD-1^+^ T cells were significantly decreased in the FIER (fibrosis and inflammation response with HBeAg seroconversion) group after treating with PEG-IFN-α for 6 months

**Fig. 2:**
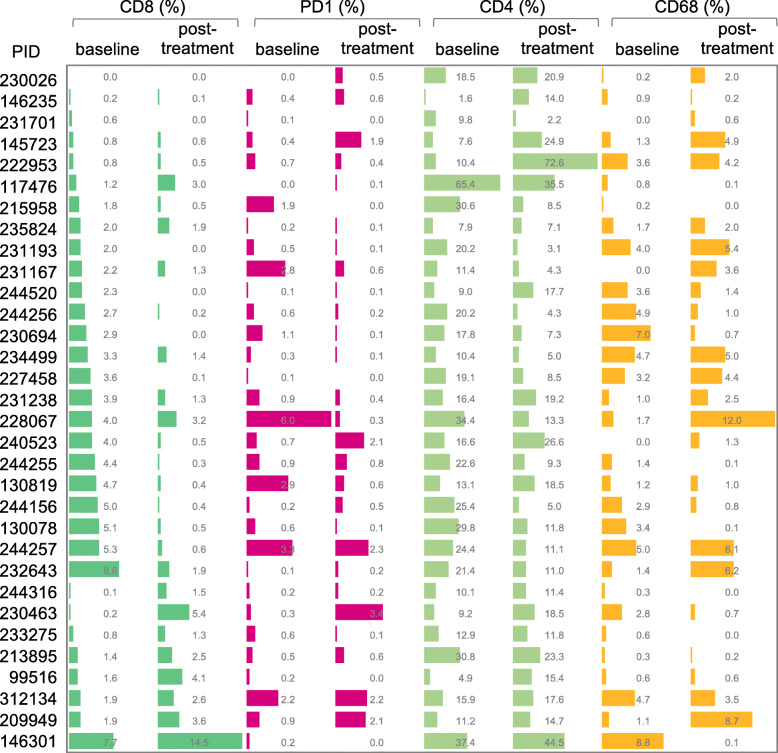
The changes in tissue infiltrating immune cells at baseline and post-treatment. The number of CD8^+^ T cells, PD-1^+^ cells, CD4^+^ T cells, and CD68^+^ mononuclear cells in 32 patients before and after 6 months’ treatment were displayed in the form of histogram and percentage figures. PID: Patient ID

The changes in the levels of CD8^**+**^ T cells, CD4^**+**^ T cells, CD68^**+**^ mononuclear cells, and PD-1^**+**^ cells before and after the therapy commencement are inconsistent among individual patients (Fig. 2). However, the overall median frequency of CD8^**+**^ T cells in the liver tissues from 32 CHB patients was significantly decreased 6 months after the initiation of PEG-IFN-α therapy (*p* < 0.01) (Table 3). The overall median frequencies of CD4^**+**^ T cells, CD68^**+**^ mononuclear cells, and PD-1^**+**^ cells decreased as well, although the differences were not significant (*p* > 0.05) (Table 3).

**Fig. 3:**
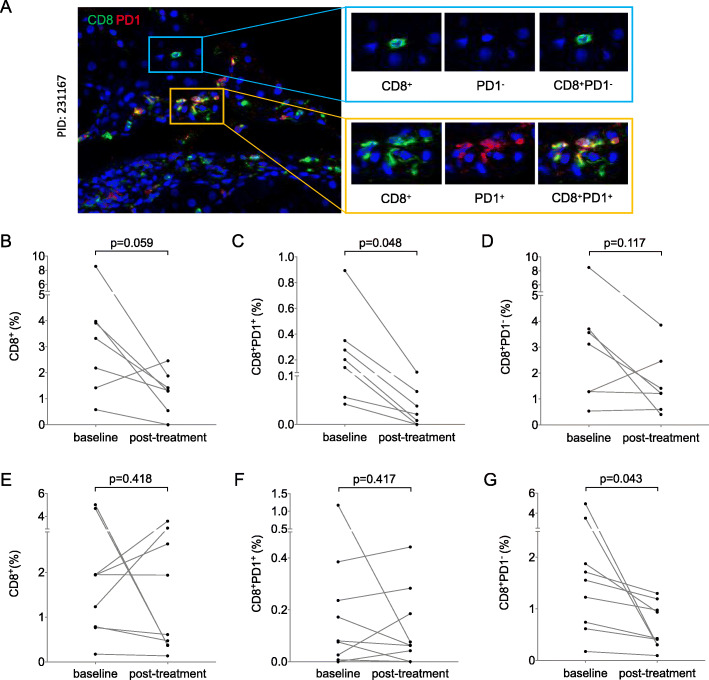
The changes in CD8^+^PD-1^+^ T cells and CD8^+^PD-1^−^ T cells at baseline and post-treatment. **a**: Representative images of CD8^+^PD-1^+^ T cells and CD8^+^PD-1^−^ T cells on the same slide from sample of case 231,167 evaluated by multiplex immunohistochemical staining. 400× magnification. PID: Patient ID. **b**-**d**: The changes in the levels of CD8^+^ T cells, CD8^+^PD-1^+^ T cells, and CD8^+^PD-1^−^ T cells at baseline and post-treatment in the FIER groups (*n* = 7). **e**-**g**: The changes in the levels of CD8^+^ T cells, CD8^+^PD-1^+^ T cells and CD8^+^PD-1^−^ T cells at baseline and post-treatment in the FIENR groups (*n* = 9)

According to the outcomes at 6 months, the patients were divided into different groups (Table 4). The FIER group and FIENR group had the largest number of cases. We used the spectral splitting and superposition techniques of inForm software to co-localize the signals of CD8 and PD-1 (Fig. 3a), and quantified the changes in CD8^+^PD-1^+^ T cells and CD8^+^PD-1^−^ T cells before and after treatment. Compared with baseline, CD8^**+**^PD-1^**+**^ T cells significantly decreased in liver tissues in the FIER group (*n* = 7) after treating with PEG-IFN-α for 6 months (Fig. 3c, *p* < 0.05), while total CD8^+^ T cells and CD8^**+**^PD-1^**−**^ T cells had no significant difference (Fig. 3b and d, *p* > 0.05). CD4^**+**^ T cells and CD68^**+**^ mononuclear cells did not change significantly in this group as well (data not shown). In the FIENR (no fibrosis and inflammation response and HBeAg seroconversion) group (*n* = 9), CD8^**+**^PD-1^**−**^ T cells in the liver tissue decreased significantly at 6 months (Fig. 3g, *p* < 0.05), while total CD8^**+**^ T cells and CD8^**+**^PD-1^**+**^ T cells had no significant difference (Fig. 3e and f, *p* > 0.05). CD4^**+**^ T cells and CD68^+^ mononuclear cells had no significant difference (data not shown).

**Table 4:** Groups by the outcomes at 6 months

Groups	Gender (male/female)	Number of patients
FR group (n)	11/3	14
IR group (n)	17/5	22
ER group (n)	9/1	10
FIER group (n)	6/1	7
FIRENR group (n)	3/2	5
IRFENR group (n)	6/2	8
FRIENR group (n)	1/0	1
FIENR group (n)	9/0	9

*FR* Fibrosis response. *IR* Inflammation response. *ER* HBeAg seroconversion, *FIER* Fibrosis and inflammation response with HBeAg seroconversion, *FIRENR* Fibrosis and inflammation response without HBeAg seroconversion, *IRFENR* Inflammation response without fibrosis response without HBeAg seroconversion, *FRIENR* Fibrosis response without inflammation response without HBeAg seroconversion, *FIENR* No fibrosis and inflammation response and HBeAg seroconversion

We also assessed the changes in the CD4^**+**^ T, CD8^**+**^ T, CD8^**+**^PD-1^**−**^ T, CD8^**+**^PD-1^**+**^ T cells, and CD68^**+**^ mononuclear cells in the FIRENR (fibrosis and inflammation response without HBeAg seroconversion) group (*n* = 5); there were no significant differences of those features between baseline and 6 months in PEG-IFN-α therapy (data not shown). In the IRFENR (inflammation response without fibrosis response without HBeAg seroconversion) group (*n* = 8), only the CD8^**+**^PD-1^**+**^ T cells decreased significantly in the portal areas after 6 months of antiviral therapy, while there was no significant difference in the lobular areas and the total (portal and lobular) areas (data not shown). There was only 1 patient in the FRIENR (fibrosis response without inflammation response without HBeAg seroconversion) group, so it could not be analyzed by statistics.

### CD68^+^ mononuclear cells increased in the FIER (fibrosis and inflammation response with HBeAg seroconversion) group after treating with PEG-IFN-α for 6 months

**Fig. 4:**
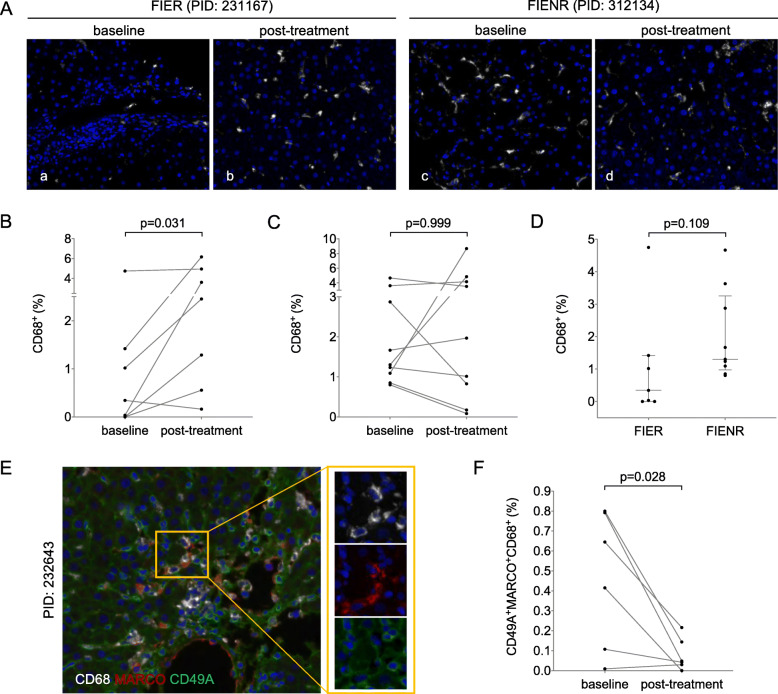
The changes in CD68^+^ mononuclear cells at baseline and post-treatment. **a**: Representative images of CD68^+^ mononuclear cells at baseline and post-treatment from samples of case 231,167 (FIER groups) and case 312,134 (FIENR groups) evaluated by multiplex immunohistochemical staining. 200× magnification. PID: Patient ID. **b**: The changes in the levels of CD68^+^ mononuclear cells at baseline and post-treatment in the FIER groups (*n* = 7). **c**: The changes in the levels of CD68^+^ mononuclear cells at baseline and post-treatment in the FIENR groups (*n* = 9). **d**: The levels of CD68^+^ mononuclear cells between the FIER groups (*n* = 7) and the FIENR groups (*n* = 9) at baseline. **e**: Representative images of CD49A^+^MARCO^+^CD68^+^ mononuclear cells at baseline from samples of case 232,643 (FIER groups) evaluated by multiplex immunohistochemical staining. 400× magnification. PID: Patient ID. **f**: The changes in the levels of CD49A^+^MARCO^+^CD68^+^ mononuclear cells at baseline and post-treatment in the FIER groups (*n* = 6). One patient’s section had been dissected during the experiment, so it was not counted

From the CD68 staining results we observed an overall inclining trend for the level of CD68^+^ mononuclear cells in some FIER patients at 6 months (Fig. 4Aa-b and b, *p* < 0.05). The levels of CD68^+^ mononuclear cells increased significantly in 6 patients in the FIER group after treatment and decreased slightly in 1 patient. However, A similar result was not observed in the FIENR group (Fig. 4Ac-d and c, *p* > 0.05), where the level of CD68^+^ mononuclear cells increased in 4 patients but decreased in the other 5 patients after treatment. We further analyzed the levels of CD68^+^ mononuclear cells between these two groups at baseline and found that although the level of CD68^+^ mononuclear cells in the FIENR group was higher than that in the FIER group, the difference was not significant (Fig. 4d, *p* > 0.05).

In order to study the cell subtypes and possible functions of these CD68^+^ mononuclear cells, we applied multiple immunohistochemistry on the same tissue section from each patient in the FIER group to stain and co-localize CD68, CD49A, and MARCO (Fig. 4e). Our results showed that the CD49A^+^MARCO^+^CD68^+^ mononuclear cells in the FIER group decreased significantly after treating with PEG-IFN-α treatment for 6 months (Fig. 4f, *p* < 0.05).

## Discussion

IFN-α has been used for many years to treat patients with chronic HBV infection, but it is unclear why some patients respond to the treatment and others do not. The immunomodulatory function of IFN-α includes the activation of NK cells and B cells and the stimulation of CD8^**+**^ T cell function both directly and indirectly [7]. Treatment with PEG-IFN-α to CHB patients can led to increased IFN-γ production from NK cells [20, 21]. IFN-α has been shown to influence the functions of both CD4^**+**^ and CD8^**+**^ T cells [8], but the observations are contradicted. It was reported that responders to IFN-α exhibited significant increases in intrahepatic CD8^**+**^ T cells [12]. The authors concluded that it is the intrahepatic CD8^**+**^ T lymphocyte response, but not CD4^**+**^ T lymphocyte or NK/NKT-cell response, that is important for HBV clearance during the IFN-α therapy [12]. In contrast, recent studies showed that PEG-IFN-α treatment resulted in a striking reduction of CD8^**+**^ T cells [10, 11]. Our research found that CD8^**+**^ T cells decreased significantly after treating with PEG-IFN-α for 6 months in 32 CHB patients, similar to the most recent reports [10, 11]. We also assessed the changes in the CD4^**+**^ T cells, however, there were no significant differences between baseline and at 6 months, which was also in agreement with a previous report [12].

Studies showed that the upregulation of PD-1 is associated with T cell exhaustion and persistent viral infection [13]. Our past study showed that in PEG-IFN-α therapy responders, PD-1 levels decreased in CD4^**+**^ and CD8^**+**^ T cells from peripheral blood, while in non-responders, CD4^**+**^PD-1^**+**^ T and CD8^**+**^PD-1^**+**^ T cells had no significant reduction [6]. In this study, CD8^**+**^PD-1^**+**^ T cells significantly decreased in liver tissue in the FIER group after treating with PEG-IFN-α for 6 months, while CD8^**+**^PD-1^**−**^ T cells did not change significantly. On the contrary, CD8^**+**^PD-1^**−**^ T cells significantly decreased in the FIENR group at 6 months, while CD8^**+**^PD-1^**+**^ T had no significant difference. This was similar to our previous results from peripheral blood and indicated that the reduction in CD8^**+**^PD-1^**+**^ T cells in liver tissue was critical for patients who responded to the PEG-IFN-α therapy.

Monocytes can bind HBV or HBV proteins, which will lead to their activation [16, 22]. In liver tissue, CD68^**+**^ mononuclear cells were regarded as KCs and liver-infiltrating monocytes/macrophages [14, 15]. NF-κB activation in CD68^**+**^ cell-enriched cell by HBV particles or HBsAg will induce the production of IL-1b, IL-6, CXCL8, and TNF, which can inhibit virus replication in primary hepatocytes [23]. Our study showed that CD68^**+**^ mononuclear cells significantly increased in the FIER group after treating with PEG-IFN-α for 6 months. This indicated that the inhibition HBV replication by PEG-IFN-α may be partly attributable to CD68^**+**^ mononuclear cells, and high levels of CD68^**+**^ mononuclear cells might be associated with fibrosis and inflammation response. In the liver, there is a group of macrophages involved in regulating the inflammatory response. This group of cells is characterized by co-expression of CD49A, VSIG4, and MARCO molecules. It is reported that the way it is participate in regulating the immune response is to secrete pro-inflammation cytokines, such as TNF-α and IL-12. Our study found that in the FIER group, although the level of CD68^+^ cells increased after 6 months in the PEG-IFN-α therapy, the level of CD49A^+^MARCO^+^CD68^+^ cells decreased [24]. This may be a factor that affects the improvement of liver inflammation.

This study also has limitations. First, the reduction in CD8^**+**^PD-1^**+**^ T cell in liver tissue during PEG-IFN-α treatment was critical to the responders, but the mechanisms need to be investigated further. Second, the study sample size was small, and a larger cohort will be necessary to validate our observations and provide more comprehensive and precise evaluations on the efficacies of PEG-IFN-α therapy. Third, we also assessed the changes in NK cells at baseline and at 6 months, but failed to obtain immunofluorescence staining results. This requires further study.

## Conclusions

In conclusion, our results indicated that CD8^**+**^PD-1^**+**^ T cell reduction in the liver tissue was critical for patients who responded to PEG-IFN-α therapy and high CD68^**+**^ mononuclear cells in the liver tissue might be associated with fibrosis and inflammation response. Monitoring the changes in CD8^**+**^PD-1^**+**^ T cells in liver tissue during antiviral therapy might be useful for predicting the outcomes of PEG-IFN-α therapy.

## Data Availability

You can contact us for supporting data at any time. The datasets used and/or analysed during the current study are available from the corresponding author on reasonable request.

## Abbreviations

CHB: Chronic hepatitis B
PEG-IFN-α: pegylated IFN-α
FIER: Fibrosis and inflammation response with HBeAg seroconversion
FIENR: No fibrosis and inflammation response and HBeAg seroconversion
HBsAg: Hepatitis B s-antigen
HCC: Hepatocellular carcinoma
TAF: Tenofovir alafenamide
TDF: Tenofovir disoproxil fumarate
ETV: Entecavir
ISGs: Interferon-stimulated genes
KCs: Kupffer cells
HBeAg: Hepatitis B e-antigen
MWT: Microwave treatment
TSA: Tyramide signal amplification
WBC: White blood cell
RBC: Red blood cell
PLT: Platelet
ALT: Alanine aminotransferase
AST: Aspartate aminotransferase
TBIL: Total bilirubin
FIRENR: Fibrosis and inflammation response without HBeAg seroconversion
IRFENR: Inflammation response without fibrosis response without HBeAg seroconversion
FRIENR: Fibrosis response without inflammation response without HBeAg seroconversion
FR: Fibrosis response
IR: Inflammation response
ER: HBeAg seroconversion
